# The role of GRP78/ATF6/IRE1 and caspase-3/Bax/Bcl2 signaling pathways in the protective effects of gallic acid against cadmium-induced liver damage in rats 

**DOI:** 10.22038/IJBMS.2023.71343.15525

**Published:** 2023

**Authors:** Volkan Gelen, Emin Sengul, Serkan Yildirim, İrfan Cinar

**Affiliations:** 1 Department of Physiology, Faculty of Veterinary Medicine, Kafkas University, Kars, Turkey; 2 Department of Physiology, Faculty of Veterinary Medicine, Atatürk University, Erzurum, Turkey; 3 Department of Pharmacology, Faculty of Medicine, Atatürk University, Erzurum, Turkey; 4 Department of Pathology, Faculty of Veterinary Medicine, Atatürk University, Erzurum, Turkey; 5 Department of Pharmacology, Faculty of Medicine, Kastamonu University, Kastamonu, Turkey

**Keywords:** Apoptosis, Cadmium, Endoplasmic reticulum – stress, Gallic acid, Hepatotoxicity, Inflammation

## Abstract

**Objective(s):**

Cadmium (CD) causes widespread and severe toxic effects on various tissues. Studies have shown that apoptosis, inflammation, and endoplasmic reticulum stress play a role in organ damage caused by CD. Phenolic compounds with strong antioxidant effects are found in various fruits and vegetables. One of these compounds is Gallic acid (GA), which is found both free and hydrolyzable in grapes, pomegranate, tea, hops, and oak bark. Result of various studies show that GA has active antioxidant, anti-inflammatory, and anti-apoptotic properties. In our study, we investigated the mechanism of the protective effect of GA on CD-induced hepatotoxicity in rats.

**Materials and Methods:**

In this study, 50 adult male Sprague Dawley rats weighing approximately 200–250 g were used and the rats were divided into 5 groups: Control, CD, GA50+CD, GA100+CD, and GA100. The rats were treated with GA (50 and 100 mg/kg body weight), and Cd (6.5 mg/kg) was administrated to the rats for 5 consecutive days. The liver enzymes, TB levels in serum samples, oxidative stress, inflammation, ER stresses, apoptosis marker, histopathology, 8-OHDG, and caspase-3 positivity were analyzed.

**Results:**

CD administration significantly increased liver enzyme levels (AST, ALT, ALP, and LDH), MDA, IL-1-β, IFN-γ, TNF-α, IL-10, IL-6, GRP78, CHOP, ATF6, p -IRE1, sXBP, Bax mRNA expression, Caspase 3, and 8-OHdG expression (*P*<0.05). These values were found to be significantly lower in the Control, GA100+CD, and GA100 groups compared to the CD group (*P*<0.05). CD administration significantly decreased the expression levels of TB, IL-4, SOD, GSH, CAT, GPX, and Bcl-2 mRNA (*P*<0.05). These values were found to be significantly higher in the Control, GA100+CD, and GA100 groups compared to the CD group (*P*<0.05).

**Conclusion:**

The results of the present study indicated that GA prevented Cd-induced hepatic oxidative stress, inflammation, ER stress, apoptosis, and tissue damage in rats.

## Introduction

Contamination of foods and animal feeds with heavy metals poses a serious global problem for human and animal health (1, 2). Cadmium (CD) has commercial importance and is widely used in the production of Nickel-CD batteries, coating, and plastic materials (3, 4, 5-9). CD, which is taken into the body, causes damage mainly to the liver and kidneys, besides accumulating in different organs (10, 11). Studies have reported that CD accumulating in the kidney and liver stimulates tissue damage (12, 13). CD causes the destruction of renal proximal tubule cells, resulting in clinical signs of glycosuria (14). In the liver, which is one of the organs that are seriously affected by CD, acute death occurs in cells due to CD accumulation (15). In addition, studies have reported that CD induces liver damage (13, 15). One of the compounds whose effects have been investigated for this purpose is phenolic compounds. Phenolic compounds with strong anti-oxidant effects are found in various fruits and vegetables (16-18). One of these compounds is Gallic acid (GA), which is found both free and hydrolyzable in grapes, pomegranate, tea, hops, and oak bark (19). Results of various studies show that GA has active anti-oxidant, anti-inflammatory, and anti-apoptotic (20-22) properties. This study planned to be carried out in line with this information, and aims to determine the protective effects of GA on oxidative stress, inflammation, ER stress, and apoptosis in CD-induced hepatotoxicity in rats and to bring them to the literature.

## Materials and Methods

In our study, the liver toxicity model induced by Cadmium chloride (CdCl2; 99.99% purity, CAS number: 655198, Sigma), (23) was studied in Sprague Dawley rats (male, weighing 220–250 g, 10–12 weeks old), and two different doses of GA (CAS number:149-91-7, Sigma) were administered to the determined groups. To obtain sufficient blood and liver tissue samples in each group, 10 rats were used and 5 groups were formed. The necessary permission for the experimental protocol of the study was obtained from Kafkas University Animal Experiments Local Ethics Committee (KAÜ-HADYEK 2022/038). The rats were housed under standard laboratory conditions (24±1 °C, 45 ± 5% humidity, and 12 hr light/dark cycle). Throughout the study, a commercial pellet diet and water were given *ad libitum*. CD and GA were dissolved in normal saline and doses of cisplatin and GA were selected based on previous studies (24, 25). The control group received 1 ml of distilled water orally for 5 days and the CD group received IP for 5 days. The CD was administered. GA50+CD and GA100+CD groups were administered oral GA at 50 and 100 mg/kg (26, 27) doses for 5 days, and 1 hr after GA administration, IP CD (6,5 mg/kg) was injected. GA group received oral GA at a dose of 100 mg/kg for 5 days. At the end of the experimental applications, after weighing the live weight of the rats, liver tissues were taken after intracardiac blood collection and cervical dislocation under sevoflurane anesthesia. After weighing these tissues, a portion of the liver tissue of 10 rats from each group was immediately taken into 10% formaldehyde after washing with saline for histopathological, immunohistochemical, and immunofluorescent examinations. The remaining part of the livers of the rats was frozen in liquid nitrogen immediately after washing with physiological saline.

***Biochemical analysis***

Aspartate transaminase (AST), alanine transaminase (ALT), alkaline phosphatase (ALP), lactate dehydrogenase (LDH), and total bilirubin (TB) levels in blood samples taken from rats were analyzed and evaluated in an autoanalyzer. Liver tissue homogenates required for oxidative stress and inflammation biomarker analyses were obtained as described in our previous study. Malondialdehyde  (MDA), glutathione (GSH) levels, superoxide dismutase (SOD), glutathione peroxidase (GPX), and catalase (CAT) activity were determined in liver tissue. In addition, interleukin-6 (IL-6), interleukin-1β (IL-1β), tumor necrosis factor-alpha (TNF-α), interleukin-4 (IL-4), and interferon gamma (IFN-γ) levels were determined. Oxidative stress parameters and cytokines in liver tissue supernatants obtained from rats were measured using a rat ELISA kit following the manufacturer’s instructions. Analyses have been carried out by an ELISA Plate Reader (Bio-Tek, Winooski, VT, USA) according to the standard manufacturer’s instructions. Absorbance was read at 450 nm.

***Gene expression analysis***

Glucose reacting protein 78 (GRP78), C/EBP homologous protein (CHOP), activating transcription factor 6 (ATAF6), inositol-requiring enzyme 1 (p-IRE1), X-box binding protein 1 (sXBP1), B-cell lymphoma protein 2-associated X (Bax), and B-cell lymphoma protein 2 (Bcl-2) mRNA expression levels in the obtained liver tissues were determined by Real-TimeTime PCR method and evaluated between groups.

**Real-time PCR analysis**

*Determination of gene expressions from tissues*

Tissues were homogenized in the Tissue Lyser II (Qiagen) device with the help of liquid nitrogen, and RNA extraction was continued in the QIAcube RNA isolation device as recommended by the manufacturer.

*Reverse transcriptase reaction and cDNA synthesis*

cDNA synthesis was performed from total RNA using the High Capacity cDNA Reverse Transcription Kit enzyme. Each reaction was performed with 10 μl of RNA and cDNA synthesis was achieved with Veriti 96 Well Thermal Cycler (Applied Biosystem) according to the following temperature values. The amount of cDNA was determined by nanodrop spectrophotometry (EPOCH Take3 Plate, Biotek) and stored at -20 °C.

*Real-time quantitative PCR*

GRP78, CHOP, ATAF6, p-IRE1, sXBP1, Bax, and Bcl-2 genes were quantified using the Taq Man Gene Expression Master Mix kit. The methods were described in our previous studies. Briefly, amplification and quantification were performed on the StepOne Plus Real-Time PCR System (Applied Biosystems). For 100 ng of cDNA, GRP78, CHOP, ATAF6, p-IRE1, sXBP1, Bax, and Bcl-2 genes and ACTB as housekeeping gene (Applied Biosystems) were pipetted as follows and run with 40 cycles. Ct values were converted to delta Ct and the findings obtained as a result of our studies were statistically evaluated in the SPSS 20.00 package program.

***Histopathological examination***

Liver tissue samples were fixed in a 10% formaldehyde solution and embedded in paraffin blocks. Sections were taken from each block, stained with hematoxylin-eosin (H&E) for histopathological examination, and examined under a light microscope.

***Immunohistochemical examination***

Tissue sections taken on adhesive (poly-L-Lysin) slides for immunoperoxidase analysis were deparaffinized and dehydrated. Then, endogenous peroxidase was inactivated by keeping it in 3% H_2_O_2_ for 10 min. Then the tissues were boiled in 1% antigen retrieval (citrate buffer (pH+6.1) 100X) solution and allowed to cool at room temperature. Sections were incubated with protein block for 5 min to prevent nonspecific background staining in tissues. Then, the primary antibody (Caspase 3 Cat No: sc-56053, Dilution Ratio: 1/100, US) was dripped onto the tissues and incubated in accordance with the instructions for use. 3-3’ Diaminobenzidine (DAB) chromogen was used as chromogen in tissues. The stained sections were examined with a light microscope (Zeiss AXIO, GERMANY).

***Immunofluorescence examination***

Tissue sections taken on the adhesive (poly-L-Lysin) slides for immunofluorescence examination were deparaffinized and dehydrated. Then, endogenous peroxidase was inactivated by keeping it in 3% H_2_O_2_ for 10 min. Next, the tissues were boiled in 1% antigen retrieval (citrate buffer (pH+6.1) 100X) solution and allowed to cool at room temperature. Sections were incubated with protein block for 5 min to prevent nonspecific background staining in tissues. Then, the primary antibody (8-OHdG, Cat No: sc-66036, Dilution Ratio: 1/100, US) was dripped onto the tissues and incubated following the instructions for use. An immunofluorescence secondary antibody was used as a secondary marker (FITC Cat No: ab6785 Diluent Ratio: 1/1000) and kept in the dark for 45 min. Afterward, DAPI with mounting medium (Cat no: D1306, Dilution Ratio: 1/200 UK) was dripped onto the sections and kept in the dark for 5 min, and the sections were covered with a coverslip. The stained tissues were examined under a fluorescent microscope (Zeiss AXIO, GERMANY).

***Statistical analysis***

In the statistical analysis of histopathological examinations, the non-parametric Kruskal-Wallis test was used to detect group interaction, and the Mann-Whitney U test was used to determine the differences between groups. Quantitative and semi-quantitative values obtained at the end of the studies were evaluated using the Tukey test after one-way ANOVA, which is used in the statistical analysis of more than two independent groups in the SPSS 20.00 statistical data program. Tukey’s test was performed to compare positive immunoreactive cells and immunopositive stained areas with healthy controls. As a result of the test, a value of *P*<0.05 was considered significant and the data were presented as mean ± SD.

## Results

***Effects of gallic acid on liver enzyme levels***

ALP, ALT, AST, and LDH levels of control, GA100+CD, and GA100 groups were normal. However, these values were quite high in the CD group. ALP and ALT enzyme levels were significantly lower in the GA100+CD group than in the CD group. AST, LDH, and TB levels were lower in the GA100+CD group than in the CD group, but there was no statistical difference (Table 1).

***Effects*** ***of gallic acid on oxidative stress***

**Figure 1:**
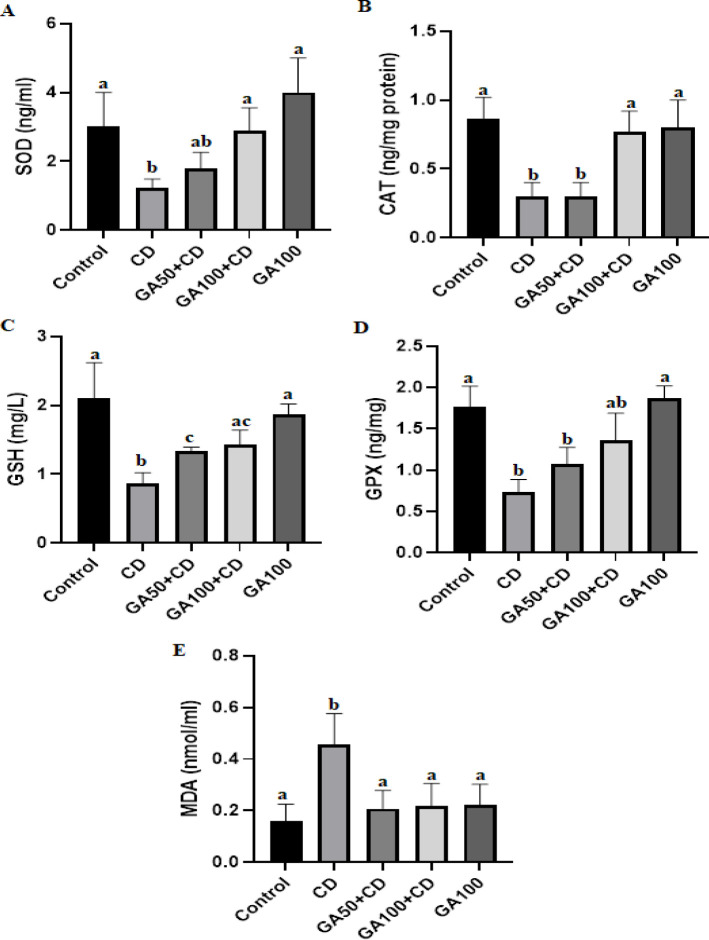
Comparison of the SOD (A), CAT (B), GSH (C), GPX (D), and MDA (E) activities/levels in the liver tissue between rat experimental groups

GSH levels and SOD, GPx, and CAT enzyme activities were decreased in the CD group. These values were normal in the control, GA50+CD, and GA100 groups. These findings showed that GA administration significantly inhibited the decrease in Cd-induced anti-oxidant enzyme activity. The MDA level in the CD group was quite high. MDA value was normal in the control, GA50+CD, and GA100 groups. These findings showed that GA administration significantly inhibited CD-induced lipid peroxidation (Figure 1).

***Effects*** ***of gallic acid on inflammation***

**Figure 2:**
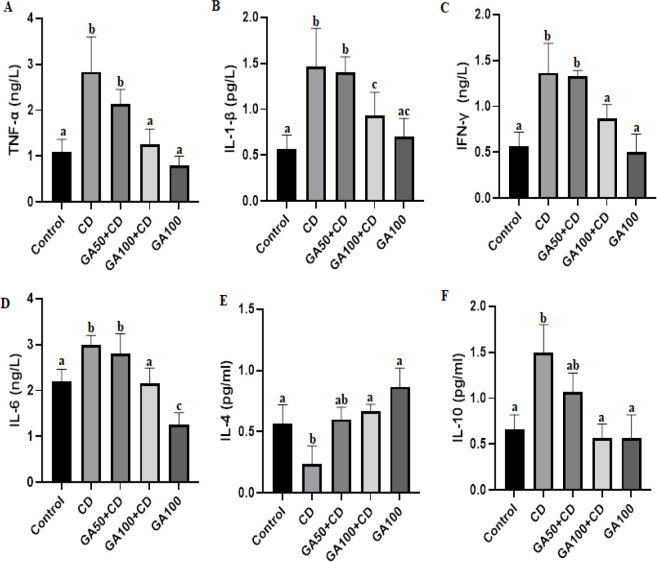
Effects of gallic acid (GA) against cadmium (CD)-induced liver inflammation in rats

IL-1-β, IFN-γ, TNF-α, IL-10, and IL-6 levels were significantly increased in the CD group. On the other hand, these values were lower in the control, GA100+CD, and GA100 groups (Figures 2A, B, C, D, and F). While 100 mg/kg dose significantly reduced the increase in GA, IL-1-β, IFN-γ, TNF-α, IL-10, and IL-6 levels, 50 mg/kg dose GA TNF-α, IL-1-β, IFN-γ, IL-10, and IL-6 did not significantly reduce levels (Figures 2A, B, C, D, and F). IL-4 levels were significantly decreased in the CD group compared to the control, GA100+CD, and GA100 groups. Low-dose GA failed to significantly prevent the reduction in IL-4 levels (Figure 2E).

***Effects*** ***of gallic acid on ER-stress***

**Figure 3:**
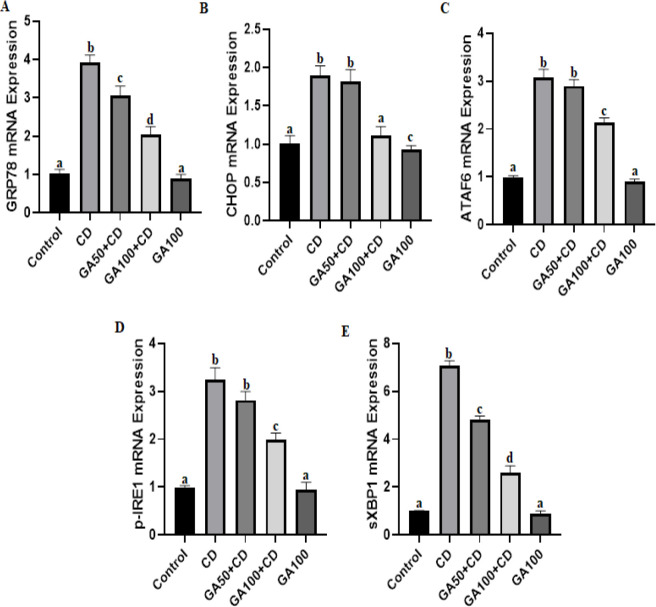
Effects of gallic acid (GA) against cadmium (CD)-induced liver endoplasmic reticulum (ER)-stress in rats

GRP78, CHOP, ATF6, p-IRE1, and sXBP1 mRNA expression levels were increased in the CD group compared to the control, GA100+CD, and GA100 groups (Figure 3). However, CHOP, ATF6, and p-IRE1 mRNA expression levels were not increased in the CD group compared to GA50+CD groups (Figure 3). This is an indication that a high dose of GA is more effective on CD-induced ER stress than a low dose.

***Effects*** ***of gallic acid on apoptosis***

**Figure 4:**
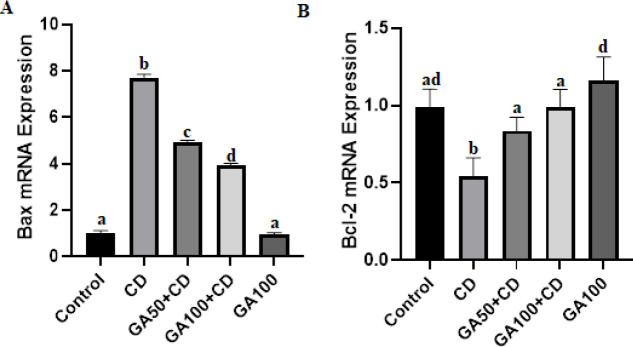
Effects of gallic acid (GA) against cadmium (CD)-induced liver ER-stress in rats

Bax mRNA expression levels increased in CD, GA50+CD, and GA100+CD groups compared to control and GA100 groups. GA prevented the increase in Bax mRNA expression levels in a dose-dependent manner (Figure 4). Bcl-2 mRNA expression level was decreased in the CD group compared the to control, GA50+CD, GA100+CD, and GA100 groups.

***Histopathological findings***

**Figure 5:**
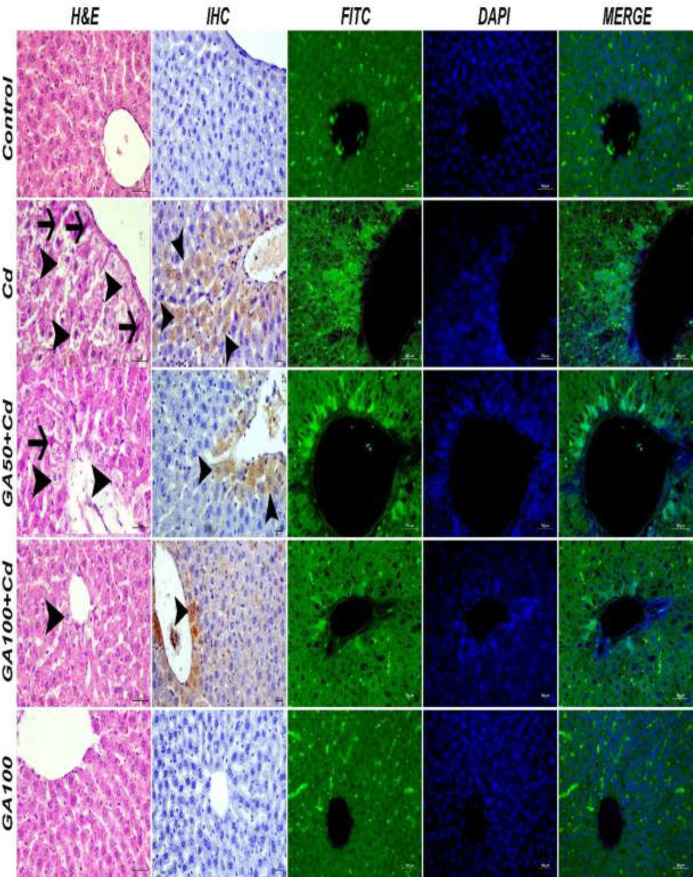
Liver tissue, hepatocytes degeneration (arrowheads), necrosis (arrows), H&E, Bar:40 µm, Caspase 3 servers (arrowheads), IHC-P, Bar:20 µm; 8-OHdG Expression (FITC, DAPI, MERGE), IF, Bar: 50 µm

It was observed that the tissues of the control and GA100 groups were in a normal structure. The CD group detected severe degeneration and necrosis in hepatocytes and hyperemia in the vessels. Moderate degeneration in hepatocytes of the acinar region, mild necrosis, and moderate hyperemia in the vessels was observed in the GA50+CD group. Mild degeneration in hepatocytes and vascular hyperemia was observed in the GA100+CD group (Figure 5). The histopathological findings are summarized in Table 2.

***Immunohistochemical findings***

Caspase-3 expression was negative in the control and GA100 groups, severe intracytoplasmic Caspase 3 was expressed in the acinar region in the CD group, moderately in the GA50+CD group, and mildly in the GA100+CD group (Figure 5). The immunohistochemical findings are summarized in Table 2.

***Immunofluorescence findings***

Expression of 8-OHdG was negative in the control and GA100 groups, intense intracytoplasmic 8-OHdG was expressed in the acinar region in the CD group, moderately in the GA50+CD group, and mildly in the GA100+CD group (Figure 5). The immunofluorescence findings are summarized in Table 3.

**Table 1:** Comparing the serum levels of liver indices, including ALP, ALT, AST, TB, and LDH between rat experimental groups (n=10)

**Groups**	**ALP (U/L)**	**ALT (U/L)**	**AST (U/L)**	**TB (mg/dL)**	**LDH (U/L)**
**Control**	82,72±15,51^a^	32,4±2,11^ a^	105,03±3,58^ a^	0,08±0,01^ a^	518,55±60,17^ a^
**CD**	171,65±17,4^b^	84,60±7,31^ b^	154,42±15,32^b^	0,06±0,01^ a^	750,15±45,83^ b^
**GA50+CD**	132,20±14,12^ bc^	67,11±7,21^bc^	167,53±20,25^ab^	0,06±0,01^ a^	692,42±67,22^ ab^
**GA100+CD**	121,40±15,2^ ac^	65,21±9,18^ ac^	123,02±7,67^ab^	0,05±0,01^a^	624,00±72,18^ ab^
**GA100**	84,12±10,31 ^a^	51,12±4,21^ a^	129,53±11,04^ a^	0,05±0,01^ a^	572,23±50,02^ a^

Values are expressed as mean ± SD. The different letters (a–c) in the lines differ statistically (*P*<0.05, n=10).

ALP: Alkaline phosphatase; ALT: Alanine transaminase; AST: Aspartate transaminase; TB: Total bilirubin; LDH: Lactate dehydrogenase

Values are expressed as mean ± SD. Different letters (a–c) in the columns differ statistically (*P*<0.05, n=10).

SODL: Superoxide dismutase; CAT: Catalase; GSH: Glutathione; GPX: Glutathione peroxidase; MDA: Malondialdehyde

Values are expressed as mean ± SD. Different letters (a–c) in the columns differ statistically (*P*<0.05, n=10).

Values are expressed as mean ± SD. Different letters (a–d) in the columns differ statistically (*P*<0.05, n=10).

Values are expressed as mean ± SD. Different letters (a–d) in the columns differ statistically (*P*<0.05, n=10).

**Table 2:** Scoring of histopathological data in liver tissues obtained from experimental groups

	Degeneration	Necrosis	Hyperemia
**Control **	-	-	-
**CD**	+++	+++	+++
**GA50+CD**	++	+	++
**GA100+CD**	+	-	+
**GA100**	-	-	-

Scored as follows: none = -; weak = +; moderate = ++; strong = +++

GA: Gallic acid; CD: Cadmium

**Table 3:** **S**coring of immunohistochemical, and immunofluorescence data in liver tissues obtained from experimental groups. Different letters (a–c) differ statistically between rat experimental groups

	**Caspase 3 Ekspresyonu**	**8-OHdG Ekspresyonu**
**Control**	19.83±0.32^a^	22.13±0.48^a^
**CD**	86.54±3.51^b^	87.59±3.16^b^
**GA50+CD**	52.16±1,98^c^	73.46±2.59^b^
**GA100+CD**	32.15±0.92^d^	34.12±0.87^c^
**GA100**	20.13±0.57^a^	21.98±0.39^a^
		

Values are expressed as mean ± SD. The different letters (a–c) in the lines differ statistically (*P*<0.05, n=10)

GA: Gallic acid; CD: Cadmium

## Discussion

CD poses a health risk to both humans and animals (28). Known sources of CD contamination; PVC products are color pigments and Ni-CD batteries (29). In areas where there is cadmium exposure, it can be transmitted to living things even through house dust (30). The transmission of CD to living things is mainly through the respiratory tract, but it can also be transmitted through the digestive tract. CD causes toxic effects (30, 31). For this reason, research on reducing CD-induced harm is of great importance. Gallic acid, one of the compounds whose effects on the effects of toxic compounds have been investigated, is a flavone (32). With this project, we aimed to determine the protective effects of GA on CD-induced liver toxicity in rats.

Serum levels of hepatic cytosolic enzymes such as ALP, LDH, AST, ALT, and bilirubin are elevated following injury to hepatocytes (33). In experimentally induced hepatotoxicity models, serum levels of these enzymes and bilirubin are significantly increased (34, 35). Akbari *et al*. (26) found that GA treatment significantly inhibited the increase in serum AST, ALT, ALP, and LDH levels in liver damage caused by acetaminophen.

Oxidative stress damages many biological molecules, especially DNA, proteins, lipids, and induces apoptosis. This damage may occur in cells by ROS (36, 37). ROS causes the oxidation of lipids in the cell membrane and membrane integrity is impaired (38). When CD accumulates in cells, intracellular oxidant and anti-oxidant balance systems are disrupted. Elimination and production of ROS are controlled under redox equilibrium. The redox homeostasis of the cells is provided by the anti-oxidant defense system of the cell. Increased ROS and oxidative stress in the cell lead to the breakdown of intracellular lipids. CD application causes the breakdown of lipids, and the level of MDA in the cell increases due to lipid peroxidation that develops as a result (39, 40). In this study, while CD application decreased GSH levels and SOD, CAT, and GPx activities in liver tissues, and increased MDA levels, GA treatment reduced these effects. Fang *et al*. (41) showed that SOD, CAT, and GPx activities and GSH levels decreased and MDA levels increased in CD liver tissue. In another study, it was found that administration of CD decreased SOD, CAT, and GPx activity, and GSH levels in liver damage (42). Previous studies have also shown that GA may have curative effects in reducing drug-induced oxidative stress in liver tissue (43, 44).

Inflammation is associated with many chronic diseases, especially cancer, diabetes, and cardiovascular and neurological diseases. However, it has been reported that various transcription factors such as AP-1, NFĸB, and p53 are activated in the case of oxidative stress (43). CD induces inflammation by increasing proinflammatory cytokine levels and decreasing anti-inflammatory cytokine levels (45). In addition, GA has been experimentally found to prevent/reduce inflammation (21, 46). 

ER is an organelle that is highly sensitive to intracellular and extracellular stimuli (47, 48). When ER stress is activated for any reason, the amount of unfolded or misfolded protein increases in the cell, and accordingly, three transmembrane proteins called p-PERK, IRE1α, and ATF6 are separated from GRP78, which combine unfolded proteins (49). Experimentally, hepatic ER stress can be induced by CD in rats (50), and expression levels of GRP78, CHOP, ATF6, p-IRE1, sXBP1, and p-PERK increase when ER stress is shaped. A study (51) found that GA reduced ER stress in diabetic hepatotoxicity in rats. In another study, it was reported that GA has a stress-reducing effect on liver toxicity (52). 

In some studies, severe histological changes have been demonstrated in the liver of rats treated with CdCl_2_ (53). On the other hand, it was reported in a study that GA application prevented cellular damage in the liver (36).

As a result of some recent studies, they reported that ROS has a very important role in stimulating the apoptotic mechanism in the cell (54). It occurs in apoptotic mechanisms by a pathway induced by members of the Bcl-2 protein family, which includes Bax and Bcl-2 (55, 56). Researchers (57) reported that in CD-induced hepatotoxicity in rats, Bax expression increased and Bcl-2 expression decreased in the toxicity group compared to other groups. In our study, CD significantly increased Bax expression in liver tissue but decreased Bcl-2 expression. In a study, it was reported that GA normalizes Bax and Bcl-2 levels in hepatocytes (58). 

Hydroxyl radicals formed as a result of oxidative stress lead to hydrogenation of nucleic acid which causes 8-OHdG (59). Various studies have shown that toxic agents increase the expression of 8-OHdG by triggering the production of ROS in liver tissue (60). 

## Conclusion

In our study, we determined that GA (especially 100 mg/kg dose) has a protective effect against oxidative stress, inflammation, apoptosis, endoplasmic reticulum stress, and DNA damage in CD-induced liver injury in rats. We think that these findings will fill the current gap in the literature and contribute to future research.

## Authors’ Contributons

V G, E Ş, S Y, and İ Ç designed and performed the research, analyzed data, and edited the article. 

## Fuding

This research has been supported by Kafkas University Scientific Research Projects Coordination Unit (Project Number: 2022-TS-45, year 2022).

## Ethical Approval

The study was approved by Kafkas University Animal Experiments Local Ethics Committee (KAÜ-HADYEK 2022/038).

## Availability of Data and Material

No additional data are available.

## Conflicts of Interest

The authors of this manuscript have no conflicts of interest to declare.
